# Genetic evidence for a species complex within the piranha *Serrasalmus maculatus* (Characiformes, Serrasalmidae) from three Neotropical river basins based on mitochondrial DNA sequences

**DOI:** 10.1590/1678-4685-GMB-2018-0131

**Published:** 2020-02-27

**Authors:** Thaís Souto Bignotto, Thiago Cintra Maniglia, Vivian Nunes Gomes, Isadora Janolio de Oliveira, Carlos Sérgio Agostinho, Sônia Maria Alves Pinto Prioli, Alberto José Prioli

**Affiliations:** 1 Universidade Estadual do Oeste do Paraná (Unioeste), Centro de Engenharias e Ciências Exatas, Grupo de Pesquisas em Recursos Pesqueiros e Limnologia (Gerpel), Toledo, PR, Brazil.; 2 Universidade Tecnológica Federal do Paraná (UTFPR), Toledo, PR, Brazil.; ^3^ Universidade Estadual de Maringá (UEM), Núcleo de Pesquisas em Limnologia, Ictiologia e Aquicultura, Maringá, PR, Brazil.; 4 Universidade Federal do Tocantins (UFT), Núcleo de Estudos Ambientais (Neamb), Porto Nacional, TO, Brazil.; 5 Universidade Estadual de Maringá (UEM), Departamento de Biotecnologia, Genética e Biologia Celular, Maringá, PR, Brazil.

**Keywords:** Cryptic species, species delimitation, D-loop, cytochrome b, cytochrome c oxidase I

## Abstract

Mitochondrial molecular markers (DNA sequences of D-loop, cytochrome b and cytochrome c oxidase I) were employed to characterize populations of the piranha *Serrasalmus maculatus* from Upper Paraná, Upper Paraguay and Tocantins River basins. D-loop sequences of *S. maculatus* population from Paraná-Paraguay River basin exhibited tandem repeats of short motifs (12 base pairs) and variable numbers depending on specimens, accounting for length variation. Concatenated mitochondrial sequences suggested that *S. maculatus* encompasses different mitochondrial DNA lineages. Although sampling was restricted to three river basins, phylogenetic analysis clearly indicated that the species currently recognized as *S. maculatus* presents high genetic variability. Maximum likelihood and Bayesian analysis clustered *S. maculatus* populations according to their locations. However, the highest genetic differentiation was identified between populations from Paraná-Paraguay system and Tocantins River basin. Three species delimitation analyses (PTP, GMYC, and ABGD) suggested that there are at least two species among the analyzed populations. The analysis of the mitochondrial sequences evidenced genetic differentiation among populations corresponding to related, but different species, suggesting that at least *S. maculatus* from the Tocantins River and Paraná-Paraguay River basins are most likely different species. Therefore, *S. maculatus* should be considered a species complex with morphologically cryptic diversity. An integrative revision is suggested.

## Introduction

For many years, piranhas have fascinated scientists around the world primarily because of their biological and evolutionary characteristics. However, the systematics of piranhas has confused ichthyologists (Fink and Machado-Allison, 2001; Jégu and Santos, 2001), and several studies revealed great ecological diversity in this group of fish (Fink and Machado-Allison, 1992; Machado-Allison and Fink, 1995a, b). Freeman *et al.* (2007) stated that the taxonomy and systematics of piranhas, as well as of other species of Serrasalmidae, Characiformes, are complex and many questions remain unanswered. *Serrasalmus* is one of the most diverse but taxonomically problematic genera of piranhas. Among the several difficulties to define species correctly, there is the probable occurrence of species complexes in the piranhas *Serrasalmus maculatus* Kner 1858 and *S. rhombeus* (L. 1766) (see for example, Freeman *et al.*, 2007).

*Serrasalmus maculatus* (Characiformes, Serrasalmidae) is a widely distributed piranha species, occurring naturally in the Amazon and Paraná-Paraguay River basins. The species had its karyotype described by specimens from different localities of the basins they occur (Cestari and Galetti Jr, 1992; Martins-Santos *et al.*, 1994; Nakayama *et al.*, 2000; Centofante *et al.*, 2002; Nakayama *et al.*, 2002, 2012). All specimens presented a diploid number of 60 chromosomes and multiple nucleolus organizer regions (NORs) located on the short arm of the acrocentric chromosomes. However, high intraspecific chromosome diversity was reported by the observation of seven distinct karyotypes, three of them occurring in the Paraná-Paraguay system and the remaining in the Amazon basin, with two of them in sympatry (Nakayama *et al.*, 2000). The presence of cytotypes found in both sympatry and allopatry for *S. maculatus* suggests that either the species presents wide karyotype heterogeneity or there is a complex of species whose morphological characteristics are very similar (Nakayama *et al.*, 2000). The high diversity of karyotypes allied to a wide geographical distribution of the species, enabled researchers to suggest that *S. maculatus* may represent a complex of cryptic species (Cestari and Galetti Jr, 1992; Nakayama *et al.*, 2000; Hubert *et al.*, 2006).

The correct species delimitation is of extreme importance not only for systematics, but also for ecology, biogeography, comparative biology, and conservation areas (Frankham *et al.*, 2012; Hernández *et al.*, 2015). Molecular methods, such as DNA sequencing, have arisen over recent decades as invaluable tools for identifying biodiversity that may not be evident by traditional morphology-based taxonomy and systematics (Bickford *et al.*, 2007; Larson *et al.*, 2016). Although molecular approaches should not replace traditional taxonomy and systematics, these methods do offer considerable power to clarify cases of convergent evolution or complex evolutionary histories (Larson *et al.*, 2016). Thus, the elucidation of the taxonomic status of *S. maculatus*, in addition to its genetic characterization, is useful to reveal intra and interspecific genetic differences, producing relevant information on biodiversity and evolutionary history of populations.

Different segments of mitochondrial DNA may be used as tools for the correct identification of species. For this reason, the aim of this study was to characterize natural populations of the *S. maculatus* piranha from Paraná-Paraguay and Tocantins hydrographic River basins using a multi-gene molecular approach [mitochondrial sequences of the control region, D-loop, and the genes cytochrome c oxidase subunit I (*coI*), and cytochrome b (*cytb*)], allied to three molecular-based species delimitation methods in order to detect the existence of a complex of cryptic species. In addition, specimens of *Serrasalmus* sp. from Tocantins River basin were included in the analysis in order to verify the genetic relationship with *S. maculatus*.

## Materials and Methods

### Sample collection

**Figure 1:**
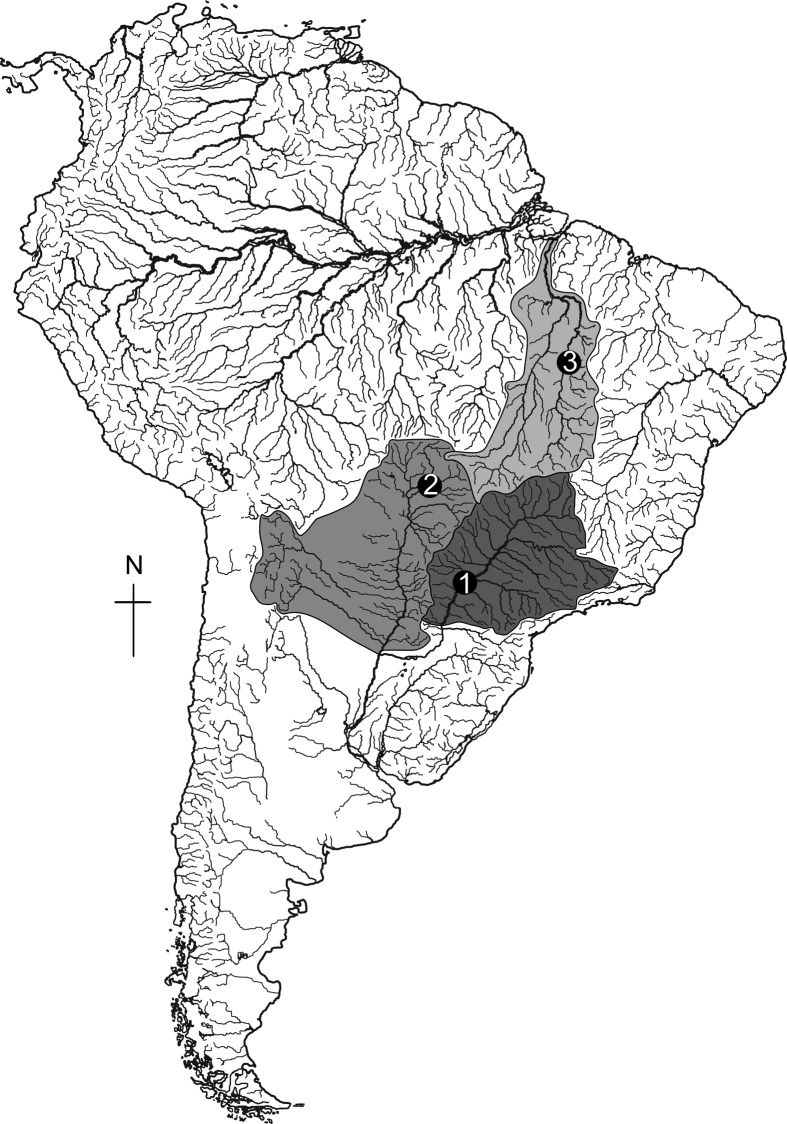
Sampling locations of *Serrasalmus maculatus* and *Serrasalmus* sp. in Brazil. Numbers correspond to the local sample: 1. Upper Paraná River basin; 2. Upper Paraguay River basin; 3. Tocantins River basin.

Four specimens of *S. maculatus* were collected in the Upper Paraná River basin (at sampling sites along the Upper Paraná River Floodplain, Baía River, and Garças lagoon), seven in the Upper Paraguay River basin (Manso River), and seven in the Tocantins River basin (Tocantins River) (Figure 1 and Table 1). Additionally, four specimens morphologically identified as *Serrasalmus* sp. of occurrence in the Tocantins River basin were collected and included in the analyses to be confronted with *S. maculatus* specimens. A sample of the red piranha *Pygocentrus nattereri* Kner 1858 was used as outgroup in the analysis. Piranha specimens of Paraná-Paraguay and Tocantins River basins were taxonomically identified by C.S. Pavanelli and C.S. Agostinho, respectively.

**Table 1:** Specimens of *Serrasalmus maculatus*, *Serrasalmus* sp. and *Pygocentrus nattereri* (outgroup) analyzed in the present study, and their respective GenBank accession numbers for mitochondrial DNA sequences of cytochrome b (*cytb*), cytochrome c oxidase I (*coI*) and control region D-loop. Code: species name and sampling sites abbreviation; N: number of analyzed specimens; Voucher: NUP – number of catalogue at Ichthyological Collection of the Nupelia (Center for Research in Limnology, Ichthyology and Aquaculture)/State University of Maringá (UEM), UNT - number of catalogue at Laboratory of Ichthyology and Systematic of Federal University of Tocantins (UFT).

Species	Code	Sampling sites	N	Voucher	GenBank Accession No.		
					*cytb*	*col*	D-loop
*S. maculatus*	SmcPY	Upper Paraguay basin (Manso River)	7	NUP 884	KP256436-442	KP256372-378	KP998542-548
*S. maculatus*	SmcPR	Upper Paraná basin (Garças Lagoon)	1	NUP 4208	KP998540	KP998541	KP998549
*S. maculatus*	SmcPR	Upper Paraná basin (Baía River)	2	NUP 4208	KP256447; 449	KP256383; 385	KP998550; 551
*S. maculatus*	SmcPR	Upper Paraná basin (Floodplain)	1	NUP 4208	KP256454	KP256390	KP998552
*S. maculatus*	SmcTO	Tocantins River	7	UNT 8175	KP256455-461	KP256391-397	KP998553-559
*Serrasalmus* sp.	SrsTO	Tocantins River	4	—	KP998566-569	KP998570-573	KP998560-563
*P. nattereri*	NtrPY	Upper Paraguay basin (Manso River)	1	NUP 886	KP256488	KP256424	KP998565

Specimens were anaesthetized and subsequently sacrificed by clove oil overdose, according to Griffiths (2000). Samples of muscle tissue were taken from each individual, fixed in commercial 96% ethyl alcohol, and kept in individual flasks. They were then stored in a freezer at -20 °C. Specimens were deposited in the Ichthyological Collection of the Center for Research in Limnology, Ichthyology and Aquaculture (Nupélia) of the State University of Maringá (UEM), and at the Laboratory of Ichthyology and Systematics of the Federal University of Tocantins (UFT) (Table 1). The study was approved by the UEM’s Committee of Ethics on Animal Care (protocol number 123/2010).

### DNA extraction, PCR amplification, and DNA sequencing

Total DNA was extracted from muscle tissue according to methodology based on phenol/chloroform (Oliveira *et al.*, 2006). After DNA quantification, fragments of the mitochondrial genome were amplified via polymerase chain reaction (PCR), from total DNA samples. PCR amplification conditions were based on Prioli *et al.* (2002). Three segments of mitochondrial DNA were used: control region (D-loop) and the cytochrome b (*cytb*) and cytochrome c oxidase subunit I (*coI*) genes. The pair of primers H16498 (5’- CCT GAA GTA GGA ACC AGA TG -3’; Meyer *et al.*, 1990) and L14841 (5’- CCA TCC AAC ATC TCA GCA TGA TGA AA-3’; Kocher *et al.*, 1989) were used to amplify by PCR a segment of almost 1,700 base pairs (bp) that included both D-loop region and *cytb* gene. However, PCR amplification and sequencing of some *S. maculatus* specimens was not possible with this set of primers. In this specific case, a second pair of primers was used, H16498 and L D-loop M (5’-WAA GCR TCG GTC TTG TAA WCC -3’, Cronin *et al.*, 1993, with modifications), resulting in a fragment ranging from 550 to 650 bp, approximately. Finally, primers H7152 (5’-CAC CTC AGG GTG TCC GAA RAA YCA RAA -3’; Ivanova *et al.*, 2007) and L6448-F1 (5’- TCA ACC AAC CAC AAA GAC ATT GGC AC -3’; Ward *et al.*, 2005) were used to amplify a partial sequence of the *coI* gene, approximately 700 bp long.

Fragments were amplified by independent PCR assays in order to be sequenced and analyzed. The reaction mix consisted of Tris-KCl buffer (20 mM Tris-HCl, pH 8.4, 50 mM KCl), 1.5 mM MgCl_2_, 2.5 μM of each primer, 0.1 mM of each dNTP, 2.5 U of *Taq* DNA polymerase, 15 ng of DNA and filtered/deionized water (Milli-Q) for a final volume of 25 μL. Amplifications of fragments were performed in a thermocycler, programmed for different temperature profiles depending on the set of primers used. The thermal profiles used for amplification of the D-loop and *cytb* regions were as follows: an initial step of 4 min at 94 °C, followed by 40 cycles of 15 s at 94 °C, 30 s at 59–61 °C, and 2 min at 72 °C, with an additional last step of 10 min at 72 °C. For the *coI* region, an initial step of 2 min at 94 °C was followed by 35 cycles of 30 s at 94 °C, 40 s at 52–55 °C, and 1 min at 72 °C, with an final step of 10 min at 72 °C.

The amplification efficiency was confirmed on 1% agarose gel. Samples were then purified with polyethylene glycol (PEG), according to Rosenthal *et al.* (1993). The purified PCR fragments were once more amplified unidirectionally with primers H16498 or L D-loop M for the D-loop region, L14841 for the *cytb* region, and L6448-F1 for the *coI* region. Approximately 50 ng of DNA from the final product of each PCR reaction were used directly in sequencing reactions with the DYEnamic ET Dye Terminator Kit (Amersham Biosciences) in a MegaBACE 1000 automatic sequencer (Amersham Biosciences) according to the manufacturer’s instructions. All sequences were deposited in GenBank (Table 1).

### Data and phylogenetic analyses

The nucleotide sequences were aligned with Clustal Omega software (Sievers *et al.*, 2011) and manually edited in the BioEdit Sequence Alignment Editor 7.0.1 (Hall, 1999). Polymorphic sites, number of parsimony informative sites, and nucleotide compositions were obtained with MEGA7 software (Kumar *et al.*, 2016). The number of haplotypes was assessed with DNAsp 6.10 software (Rozas *et al.*, 2017). Fixation indices (*F*_*ST*_) and analysis of molecular variance, AMOVA, were calculated using Arlequin 3.11 (Excoffier *et al.*, 2005).

Three independent single-gene phylogenies were constructed using the D-loop, *coI*, and *cytb* mtDNA regions, excluding any redundant sequences. After checking for congruence among tree topologies derived from the single-gene phylogenies, analyses were based on the concatenated sequences of the D-loop, *coI*, and *cytb* sequences, resulting in a single large alignment. The best-fit model of nucleotide evolution was estimated by PartitionFinder 2.1 software (Lanfear *et al.*, 2012). Phylogenetic analyses were based on both maximum likelihood (ML) and Bayesian (BA) approaches. First and second codon positions of the coding regions *coI* and *cytb*, and the third codon position of the *coI* and *cytb* together with the noncoding D-loop region, were used as two different partitions in the concatenated analyses, as defined by the PartitionFinder 2.1.

Best-scoring ML trees were estimated for each dataset using the raxmlGUI software (Silvestro and Michalak, 2012), using rapid bootstrap algorithm, autoMRE function for resamplings, and the substitution model and partition set, previously defined by the PartitionFinder 2.1. Bayesian trees were calculated using the uncorrelated lognormal relaxed-clock model implemented in BEAST 1.8.2 with an input file generated in BEAUti 1.8.0 (Drummond *et al.*, 2012). The Yule process of speciation, which assumes a constant speciation rate among lineages, was applied as a tree prior. Each analysis ran for 10,000,000 generations with a sample frequency of 1,000. The final trees were calculated after 10% of burn-in. Length of burn-in was determined by examination of traces in Tracer 1.6 (Rambaut *et al.*, 2014). Support for nodes was determined using posterior probabilities (PP; calculated by BEAST).[Bibr B57]

### Species delimitation

Three methods for species delimitation were used to identify the specific boundaries in *S. maculatus* and *Serrasalmus* sp.: (i) the Poisson tree process model (PTP; Zhang *et al.*, 2013); (ii) the General Mixed Yule Coalescent method (GMYC, single and multiple threshold algorithm) of Pons *et al.* (2006), and (iii) the Automated Barcode Gap Discovery (ABGD) method of Puillandre *et al.* (2012). These methods were applied to unique haplotypes (redundant sequences were excluded) for the *coI* dataset only, in order to preserve consistence in the relation of this fragment and to specify Molecular Operational Taxonomic Units (MOTUs) (i.e., DNA barcoding; Hebert *et al.*, 2003; Larson *et al.*, 2016). Unlike ABGD that uses detection of the `barcode gap’ in the distribution of genetic pairwise distances, GMYC and PTP use a phylogenetic input tree from which the fit of speciation and coalescent processes are modeled to delineate MOTUs (Tang *et al.*, 2014; Larson *et al.*, 2016).

The ABGD method was conducted on the online server http://wwwabi.snv.jussieu.fr/public/abgd with the default parameters and the Kimura model (K80) of nucleotide substitution. The PTP model was implemented on the server http://species.h-its.org using the best-scoring ML tree constructed with the raxmlGUI, via the aforementioned protocol. The GMYC was implemented using the ultrametric tree based on the Bayesian inference constructed in BEAST, as mentioned above. Tracer 1.6 software (Rambaut *et al.*, 2014) was used to check for chain convergence and the effective sampling size (ESS > 200). The identification of significant clusters was implemented in RStudio software (2016) by using the *splits* package (Ezard, 2009). K2P distance based on *coI* sequences within and between principal clusters defined by PTP, ABGD, and GMYC species delimitation methods were obtained with MEGA7.

## Results

### Data and phylogenetic analyses of mitochondrial DNA sequences

**Figure 2:**
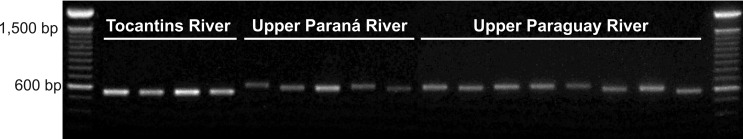
PCR amplification products of the mitochondrial DNA hypervariable region (control region, D-loop) evidencing the length polymorphism in populations of *Serrasalmus maculatus* from Tocantins, Upper Paraná, and Upper Paraguay River basins. First and last columns contain the standard molecular size ladder 100 bp (Invitrogen).

PCR amplification of the D-loop region, with primers H16498 and L D-loop M, resulted in fragments of different sizes, ranging from 550 to 650 bp, approximately (Figure 2). *Serrasalmus maculatus* population from Tocantins River basin presented the smallest fragments, with approximately 550 bp, while individuals from Upper Paraná and Upper Paraguay River basins presented fragments between 580 bp and 650 bp, characterizing a length polymorphism of the D-loop region. The observed size variation for the D-loop region in *S. maculatus* was mainly due to tandem repeats identified at the 5’ extremity of the D-loop (H-strand), nearby the tRNA^Pro^ gene, and were exclusive to *S. maculatus* population from Paraná-Paraguay system (Table 2). Thus, the presence or absence of these repetitive regions in the D-loop enabled the characterization of *S. maculatus* populations of Paraná-Paraguay River basins or Tocantins River, respectively. Repeated motifs had 12 bp in size and three to five repetitions (Table 2).

**Table 2:** Tandem repeats observed on the D-loop mitochondrial region of *Serrasalmus maculatus* populations from the Upper Paraguay (SmcPY) and Upper Paraná (SmcPR) River basins. Letters in bold highlight differences among specimens.

Sample	Repetitive motifs	Number of repetitions	Length of Tandem Repetition Region
SmcPY51, 52, 58	GGC**A**CCCCACA**T**	4	48 bp
SmcPY53, 61	GGC**A**CCCCACA**T**	5	60 bp
SmcPY55	GGC**A**CCCCACA**C**	5	60 bp
SmcPY69	GGC**A**CCCCACA**T**	3	36 bp
SmcPR12, 04	GGC**G**CCCCACA**T**	5	60 bp
SmcPR08, 15	GGC**G**CCCCACA**T**	4	48 bp

After sequence editing and trimming, partial sequences of *coI* (548 bp), *cytb* (592 bp), and D-loop (369 to 414 bp) were concatenated, resulting in an alignment of 1,509 to 1,554 bp in length. Considering *S. maculatus* and *Serrasalmus* sp. (excluding outgroup), 120 variable sites were identified (30 in *coI*, 28 in *cytb*, and 62 in D-loop), containing 102 parsimony-informative (27 in *coI*, 24 in *cytb*, and 51 in D-loop), besides several indels (insertions/deletions) occurring only in the D-loop region. The nucleotide frequencies in the in-group were A = 26.4%, T = 26.4%, C = 30.8%, and G = 16.4%. Sixteen haplotypes were identified in the concatenated sequences (9 in *coI*, 8 in *cytb*, and 16 in D-loop). Only one haplotype (Hap_12) was common to different specimens of *S. maculatus* (samples SmcTO 65, 69, 70, 73, 74, 80) and *Serrasalmus* sp. (sample SrsTO 60), both from Tocantins River basin, i.e., these species shared a mitochondrial haplotype.

Most of the observed genetic variability (88.96%) was found among populations of *S. maculatus* and *Serrasalmus* sp. from the Upper Paraná, Upper Paraguay, and Tocantins River basins, according to results of AMOVA (Table 3). Fixation indices *F*_*ST*_ revealed that the three populations of *S. maculatus* differed from each other (Table 4). It is evident that both *S. maculatus* and *Serrasalmus* sp. from Tocantins River basin are distinct in comparison to *S. maculatus* populations from Upper Paraná and Upper Paraguay River basins due to values of *F*_*ST*_ higher than 0.879. On the other hand, *Serrasalmus* sp. was not differentiated from *S. maculatus* population from the Tocantins River, with *F*_*ST*_ = 0.097.

**Table 3:** Analysis of molecular variance (AMOVA) for populations of *Serrasalmus maculatus* and *Serrasalmus* sp. from Paraná-Paraguay and Tocantins River basins.

Variation source	df	Sum of squares	Components of variation	Percentage of variation
Between populations	3	646.977	39.299	88.96^*^
Within populations	18	87.750	4.875	11.04
Total	21	734.727	44.174	
Fixation index (*F*_*ST*_) = 0.890				

* *P* < 0.01

**Table 4:** Fixation index *F*_*ST*_ (below diagonal) obtained by the concatenated mitochondrial sequences (cytochrome c Oxidase I, cytochrome b and D-loop) and mean values of K2P distances obtained from the Cytochrome c Oxidase I gene between and within (above and diagonal) populations of *Serrasalmus maculatus* and *Serrasalmus* sp. from the Upper Paraná (PR), Upper Paraguay (PY) and Tocantins (TO) River basins.

	1	2	3	4
**1.** *S. maculatus* (PY)	0.34%	1.73%	3.77%	3.77%
**2.** *S. maculatus* (PR)	0.676^*^	0.12%	4.66%	4.66%
**3.** *S. maculatus* (TO)	0.912^*^	0.953^*^	0.00%	0.00%
**4.** *Serrasalmus* sp. (TO)	0.879^*^	0.923^*^	0.097^ns^	0.00%

* *P* < 0.05; ^ns^ *P* > 0.05

**Figure 3:**
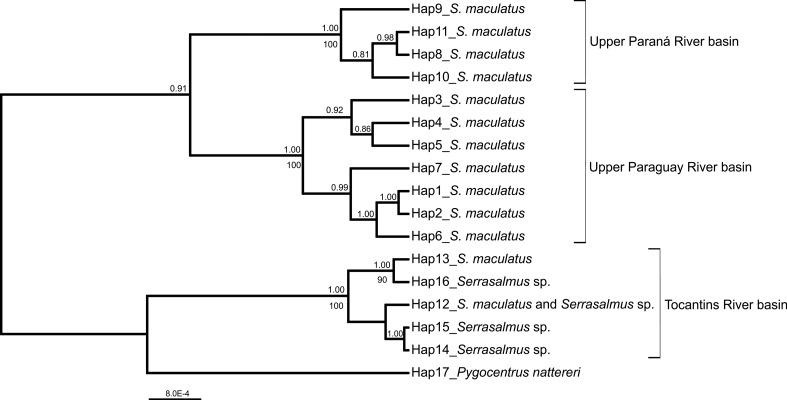
Bayesian phylogenetic tree for *Serrasalmus maculatus* and *Serrasalmus* sp. from Paraná-Paraguay and Tocantins River basins based on concatenated nucleotide sequences of the mitochondrial regions cytochrome c oxidase I, cytochrome b and D-loop. Values near branches indicate Bayesian (posterior probability, PP; above) and maximum likelihood (bootstrap; below) support values for each node. Sixteen haplotypes (Hap) were recovered for *Serrasalmus* specimens. *Pygocentrus nattereri* (Hap17) was included as outgroup.

ML and BA phylogenetic trees were congruent regarding the formation of the haplogroups of *S. maculatus* and *Serrasalmus* sp. based on the concatenated sequences (Figure 3) and each of the single gene/region analyses (Figure S1). Most of the results indicated *S. maculatus*, as well as *Serrasalmus* sp., as a monophyletic group, except in the BA analyses using the concatenated sequences and the D-loop region. The clustering of *S. maculatus* specimens according to their localities was supported by high bootstrap and posterior probability values in the ML and BA analysis, respectively. Genetic differentiation between populations from Paraná-Paraguay system and Tocantins River was evident. *Serrasalmus maculatus* populations from the Upper Paraná River basin and the Upper Paraguay River were also separated in the dendrograms, but to a lesser degree. Specimens of *Serrasalmus* sp. were grouped in the same clade of *S. maculatus* from Tocantins River, with no genetic differentiation among these species, since there is haplotype sharing as previously described.

### Species delimitation

**Figure 4:**
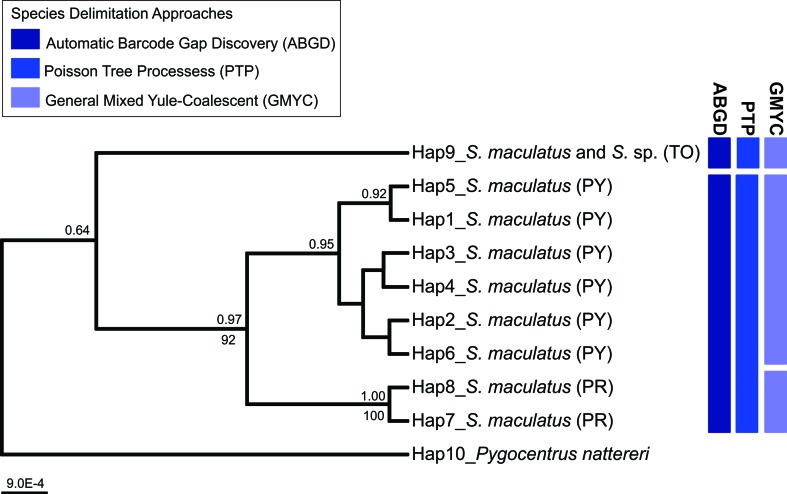
Species delimitation analyses based on the cytochrome c oxidase I (*coI*) sequences of *Serrasalmus maculatus* and *Serrasalmus* sp. from Upper Paraná (PR), Upper Paraguay (PY), and Tocantins (TO) River basins, using Automatic Barcode Gap Discovery (ABGD), Poisson Tree Processes (PTP), and General Mixed Yule Coalescent (GMYC) methods. Bayesian (posterior probability, PP; above) and maximum likelihood (bootstrap; below) support values for each node in the Bayesian phylogenetic *coI* tree. Nine haplotypes (Hap) were recovered for *Serrasalmus* specimens. *Pygocentrus nattereri* (Hap10) was used as outgroup.

Results of ABGD, PTP, and GMYC obtained with *coI* sequences are summarized in Figure 4. Both ABGD and PTP methods resulted in the delimitation of two MOTUs, whereas GMYC recovered three MOTUs. In the first case, groups were defined as: (1) specimens of *S. maculatus* and *Serrasalmus* sp. from Tocatins River basin, and (2) specimens of *S. maculatus* from the Paraná-Paraguay system. The three MOTUs defined by GMYC approach included: (1) specimens of *S. maculatus* and *Serrasalmus* sp. from the Tocantins River, (2) specimens of *S. maculatus* from the Upper Paraná River, and (3) *S. maculatus* from the Upper Paraguay River.

The mean values of inferred genetic distance based on the K2P model were also sufficient to discriminate populations (Table 4). Within each population, these values were low (ranging from 0 to 0.34%), but at least five times higher when comparing the genetic distances between populations (from 1.73 to 4.66%; see Table 4). Populations from the Upper Paraná River and the Upper Paraguay River were different based on low K2P distance (1.73%). However, when comparing populations of *S. maculatus* from Paraná-Paraguay system with those from Tocantins River, the genetic differentiation was higher, ranging from 3.77 to 4.66%. The same values were observed between *Serrasalmus* sp. and *S. maculatus* from Paraná-Paraguay system. Moreover, no genetic differentiation was detected between *Serrasalmus* sp. and *S. maculatus* both from Tocantins River (0.00%) (Table 4).

## Discussion

### Phylogenetic analyses

*Serrasalmus maculatus* is a piranha species broadly distributed throughout the Amazon and Paraná-Paraguay rivers. In addition to this extensive geographic distribution, the species presents pronounced karyotypic variability (Cestari and Galetti Jr, 1992; Martins-Santos *et al.*, 1994; Nakayama *et al.*, 2000; Centofante *et al.*, 2002; Nakayama *et al.*, 2002). The cytotypes found in this species are associated with their hydrographic basins, characterizing differences among populations, which allows the assumption that *S. maculatus* may actually comprise a complex of cryptic species (Cestari and Galetti Jr, 1992; Nakayama *et al.*, 2000; Hubert *et al.*, 2006). Similarly, our phylogenetic analyses provided further evidence that *S. maculatus* includes morphologically cryptic diversity.

Our results were also in agreement with the karyotype differences previously reported between populations of *S. maculatus* from the Amazon basin and Paraná-Paraguay system (Nakayama *et al.*, 2000, 2002; Centofante *et al.*, 2002). The mitochondrial molecular markers used in this study were efficient in recognizing the wide genetic differentiation between *S. maculatus* populations of Tocantins River and Paraná-Paraguay system. Genetic distance (K2P), *F*_*ST*_ values, and species delimitation results were consistent in order to demonstrate the genetic differentiation of these two populations.

The genetic differentiation between populations of *S. maculatus* from the Tocantins River and the Paraná-Paraguay system was also revealed by characteristics found specifically in the mitochondrial DNA control region (D-loop). In this segment, a complex pattern of variation involving several indels and tandem repeats were also identified. The majority of indels enabled the characterization of populations, but the sharper difference was observe when *S. maculatus* from Tocantins River was compared with the population of the Paraná-Paraguay system (data not presented). Tandem repetitions were also identified in the D-loop sequences, evidencing again the distinctiveness of the populations, since these repetitions were present exclusively in *S. maculatu*s from the Paraná-Paraguay system. Ortí *et al.* (2008) also detected the same type of repetitions in a specimen of *S. maculatus* from the Uruguay River. These tandem repeats typically occur at the 5’ or 3’ extremities of the D-loop, where replication of the mtDNA begins or ends, respectively (Nesbo *et al.*, 1999), and may be related to size variations within and between individuals or species (White and Martin, 2009).

Our results indicate that there is a limited connectivity between populations of *S. maculatus* of the Tocantins River and Paraná-Paraguay system, suggesting that they are evolutionarily independent lineages. The isolation of *S. maculatus* populations may have started with the formation of the Amazon and Paraná-Paraguay basins, 10 million years ago (Ma) (Hubert and Renno, 2006), by vicariance. However, Lundberg *et al.* (1998) reported that headwater catchments of the Paraná system by the Amazon system continued even after the establishment of these two basins. Montoya-Burgos (2003) and Hubert and Renno (2006) also identified probable routes of dispersion between the Madeira and Guaporé River basins and the Paraguay River. It is possible that presently there still exists a communication between basins during rainy periods, with a consequent exchange of faunas in the region. Several studies confirm the possibility of connection between rivers of the Amazon and Paraná-Paraguay basins (Garda and Cannatella, 2007; Antunes *et al.*, 2010; Aquino and Schaefer, 2010). Consequently, both dispersion and vicariance events may have influenced the differentiation of *S. maculatus* populations. According to the values of genetic divergence observed, it seems more plausible that populations have interrupted gene flow at a time following the separation of these two hydrographic basins.

The difference between *S. maculatus* populations of the Upper Paraguay River and Upper Paraná River was observed both at cytogenetic (Cestari and Galetti Jr, 1992) and molecular levels (present study). Additionally, Machado *et al.* (2018) refuted the monophyly of *S. maculatus* and reported that the population of *S. maculatus* from the upper Paraná River formed a distinct lineage from the population of the lower Paraná River. Similarly, in the present study, the two populations were genetically differentiated from each other; nevertheless, with relatively low values. In addition, the presence of regions containing tandem repeats in the D-loop sequences, presented only in the *S. maculatus* specimens of the Upper Paraná and Upper Paraguay Rivers, suggests that these populations had a common origin. Previous to the construction of the Itaipu reservoir in 1982, the Upper Paraná River was isolated from the rest of the Paraná-Paraguay system by the Sete Quedas falls, a natural geographic barrier that prevented the free dispersion of fish, mainly upstream (Menezes, 1972). Although, considering the downstream course, migration of individuals may occur with some frequency. Therefore, two events could explain the low values of genetic differentiation observed among the *S. maculatus* populations from the Upper Paraná River and the Upper Paraguay River: eventual downstream migrations and the incorporation of the subpopulation from the Itaipu region to the Upper Paraná River population.

*Serrasalmus* species occurring in the Tocantins River basin (*S. maculatus* and *Serrasalmus* sp.; Lucinda *et al.*, 2007) presented no significant difference based on the mtDNA sequences investigated in this study. Although *S. maculatus* and *Serrasalmus* sp. from Tocantins River are morphologically different from each other, mainly considering the color pattern, both species demonstrated shared mitochondrial haplotype, resulting in a single clade in dendrograms; besides, they presented low *F*_*ST*_ index and no K2P genetic distance, indicating that the taxonomic validity of these species in the Tocantins River basin should be revised. One possibility would be that lineages did not have sufficient time to reach the condition of evolutionarily independent entities. Alternatively, hybridization events, with *S. maculatus* as the maternal parent, may be promoting genetic homogenization of the two populations; or even, the combination of these factors.

Nuclear molecular markers should be used to indicate the occurrence of interspecific hybrids between *S. maculatus* and *Serrasalmus* sp. in the Tocantins River basin. Although there are no reports on the occurrence of interspecific natural hybrids of piranhas in the literature, Hubert *et al.* (2008) obtained evidence of former introgressions followed by hybridization between *Serrasalmus* sp. and *S. compressus*, and between *Serrasalmus* sp. and *S. hollandi*, sympatric species of the Upper Madeira River. Under these circumstances, a taxonomic revision of *Serrasalmus* sp. and *S. maculatus* from the Tocantins River basin is suggested.

### Species delimitation

The three analyses of species delimitation based on *coI* sequences (ABGD, PTP, and GMYC) presented two possible scenarios for limits of species in *S. maculatus* and *Serrasalmus* sp.: i) each of the three populations comprises a different species or, ii) at least the population from Tocantins River and the population from Paraná-Paraguay system belonging to two distinct species. Although the methods used to delimit species did not achieve consensus for the numbers of MOTUs (ABGD and PTP = 2 MOTUs; GMYC = 3 MOTUs), the results are not in conflict, since the divisions among MOTUs defined by ABGD and PTP were also recovered by GMYC.

Differences in mitochondrial nucleotide sequences have been used for distinguishing species for more than 30 years (Avise, 2004). Hebert *et al.* (2003) suggested that a region of the *coI* gene is appropriate as a tool in the identification of animals at the species level (DNA barcode). A standard sequence threshold of 10× the mean intraspecific variation for the group under study was proposed (Hebert *et al.*, 2004). Consequently, a divergence 10× greater than the mean of the intraspecific variation would be indicative of a new species. With this limit applied to our data (0.15% average intraspecific variation), the 10× threshold (1.53%) would establish each of the three *S. maculatus* populations analyzed as distinct species (see Table 4).

Phylogenetic analysis, in combination with estimates of species delimitation, suggests that *S. maculatus* includes morphologically cryptic diversity. The data obtained in this study strongly indicate that the populations currently identified as *S. maculatus* from the Paraná-Paraguay and Tocantins River basins are different species. The differences highlight that populations of *S. maculatus* remained isolated geographically long enough for speciation to occur. Thus, there is strong evidence that *S. maculatus* constitutes a complex of at least two morphologically similar species in the hydrographic basins of the Tocantins and Paraná-Paraguay Rivers. Since the type-locality of *S. maculatus* is the Guaporé River basin (Jégu and Santos, 2001), it would be necessary the inclusion of specimens of this basin in future studies to determine if the denomination *S. maculatus* should be restricted to the Tocantins River basin or to the Paraná-Paraguay system. Therefore, *S. maculatus* should be treated as a complex of species distributed in several regions of South America. Increasing sampling efforts of populations from other hydrographic basins could reveal other haplogroups corresponding to new species. It is possible that populations currently identified as *S. maculatus* comprise a complex of several species.

## Supplementary material

The following online material is available for this article:

Figure S1 Individual Bayesian phylogenetic trees for *Serrasalmus maculatus* and *Serrasalmus* sp. from Upper Paraná (PR), Upper Paraguay (PY), and Tocantins (TO) River basins based on nucleotide sequences of the mitochondrial regions Cytochrome c oxidase I (*coI*), Cytochrome b (*cytb*) and control region (D-loop).
