# Supplementary material for: Genetic evidence for a species complex within the piranha Serrasalmus maculatus (Characiformes, Serrasalmidae) from three Neotropical river basins based on mitochondrial DNA sequences
Source: Genet Mol Biol. 2020 Feb 27;43(1):e20190131. doi: 10.1590/1678-4685-GMB-2018-0131 (PMC7231549; doi:10.1590/1678-4685-GMB-2018-0131)
Supplement: Supplementary file 1 [file 1415-4757-GMB-43-1-e20180131-s001.pdf]

Supplementary Material to "Genetic evidence for a species complex within the piranha *Serrasalmus maculatus* (Characiformes, Serrasalmidae) from three Neotropical river basins based on mitochondrial DNA sequences"

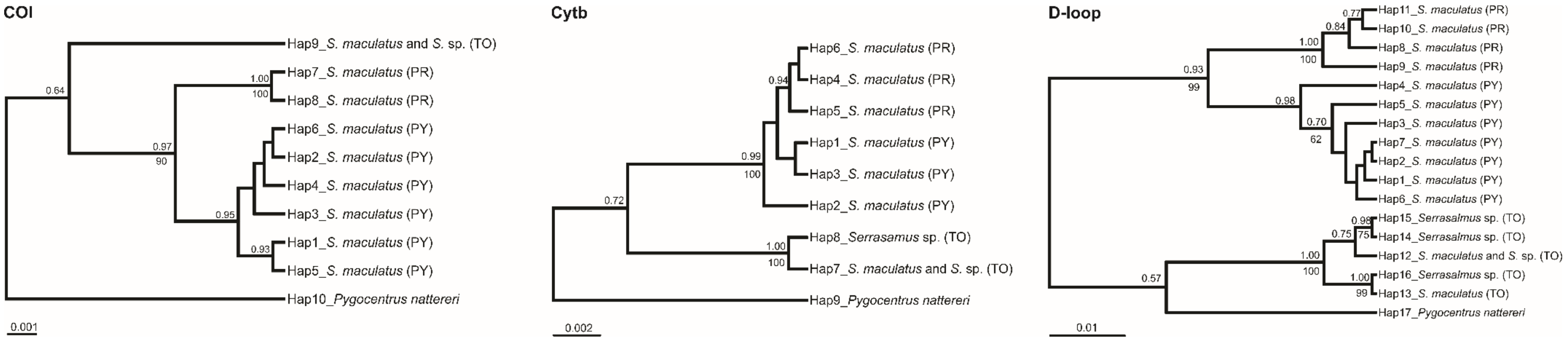

Figure S1 - Individual Bayesian phylogenetic trees for *Serrasalmus maculatus* and *Serrasalmus* sp. from Upper Paraná (PR), Upper Paraguay (PY), and Tocantins (TO) River basins based on nucleotide sequences of the mitochondrial regions Cytochrome c oxidase I (*coI*), Cytochrome b (*cytb*) and control region (D-loop). Values near branches indicate Bayesian (posterior probability, PP; above) and maximum likelihood (bootstrap; below) support values for each node. *Pygocentrus nattereri* was used as outgroup in all analysis. Hap: haplotypes observed for *Serrasalmus* (and *Pygocentrus*) specimens.
